# GitLab: work where you want, when you want

**DOI:** 10.1186/s41469-020-00087-8

**Published:** 2020-11-16

**Authors:** Prithwiraj Choudhury, Kevin Crowston, Linus Dahlander, Marco S. Minervini, Sumita Raghuram

**Affiliations:** 1 Harvard Business School, Boston, MA 02163 USA; 2 Syracuse University, Syracuse, NY 13244-4100 USA; 3 ESMT Berlin, Schlossplatz 1, 10178 Berlin, Germany; 4 INSEAD Business School, 1 Ayer Rajah Ave, Singapore, 138676 Singapore; 5 San Jose State University, One Washingon Square, San Jose, CA 95192-0070 USA

**Keywords:** Organizational design, New forms of organizing, Remote work, All remote, Virtual organizations, COVID-19

## Abstract

GitLab is a software company that works “all remote” at the scale of more than 1000 employees located in more than 60 countries. GitLab has no physical office and its employees can work from anywhere they choose. Any step of the organizational life of a GitLab employee (e.g., hiring, onboarding and firing) is performed remotely, except for a yearly companywide gathering. GitLab strongly relies on asynchronous coordination, allowing employees to work anytime they want. After highlighting some of the main practices implemented by GitLab to effectively work all remotely and asynchronously, I asked renowned organizational scientists their thoughts on this interesting case and to question the generalizability of the all remote asynchronous model. Understanding whether and under what conditions this model can succeed can be of guidance for organizational designers that are now considering different remote models in response of the COVID-19 shock and its aftermath.

## Overview of GitLab

### Marco Minervini

Remote working (also known as distributed/virtual working or tele-commuting) is not new. Last year in the UK, more than 1.5 million people worked from home full-time, nearly twice as many as 10 years ago (UK Office for National Statistics 2018), and 43% of all U.S. employees in 2016 reported working off-site at least part of the time (Gallup 2017). Another report stated that up to 70% of global professionals telecommute at least 1 day a week (International Workplace Group 2018). This is not to say it lacked critics: in the U.S. the Trump administration reversed teleworking policies that had been given a boost under the Obama administration, and prominent corporations such as Yahoo and IBM called their employees back to the office to produce synergies from collaboration (Rein 2020).

The COVID-19 pandemic created a shock of unprecedented scale to organization designs in the first quarter of 2020, in that remote working went from being a perk for a few or the privilege of all employees at a handful of start-ups, to an urgent necessity to combat the spread of viral infection. In this context, as organizations struggle to adapt to remote working, the organization design of GitLab repays close attention.

#### Background

GitLab was incorporated in 2014 and operates in the software development tools industry. In September 2019, after its Series E round of funding, GitLab was valued at $2.7 billion.

GitLab is an “all remote” company, in that all 1000+ company employees located in 60+ different countries work remotely and typically asynchronously, often without ever coming into contact with each other in the physical world. The organization has expanded significantly in 2019 from about 374 employees just a year ago.^1^

GitLab develops tools that allow software engineers to automate many parts of the software development cycle—from initial planning to final deployment and monitoring of new code in use.^2^ It is widely recognized for its “continuous integration” (CI) product,^3^ which enables teams of coders to slice a complex project into chunks, work in parallel on specialized tasks, then put the pieces back together again into a functioning whole. Specifically, GitLab’s CI tools automates verification of compatibility of new contributed code to the existing code base. They thus represented the automation of the coordination work previously conducted by a human coder.

Apart from its “all remote” model, GitLab is noteworthy to organization designers for at least three other reasons. First, like many technology companies, it uses its own tools—GitLab (the company) uses GitLab (the product) to make improvements in GitLab (the product). Second, it also uses the same set of tools to organize and manage itself—for example, the company handbook^4^ which exhaustively documents its formal organizational structure and processes, is itself developed, maintained and edited as if it were a code repository. Third, the handbook is public; anybody can view it inside or outside the company.

#### “All remote” at GitLab

GitLab’s instantiation of remote working is quite extreme—GitLab has no headquarters (except for postal and legal purposes) or offices for its employees, so they can work from anywhere they choose. Employees at all hierarchical levels work remotely, even managers at C-levels. Furthermore, GitLab has employees across almost all time zones across the globe, and it has no predetermined working hours. GitLab employees are free to determine the time of the day they prefer to work. Even hiring, onboarding and firing at GitLab takes place remotely. The only instance where all GitLab employees can be co-located is in the yearly “Contribute” event, a mostly informal corporate get-together for GitLab team members to “get face-time with one another, build community and reinforce cultural values.”^5^

Another unique aspect of GitLab’s approach to remote working is its heavy reliance on asynchronous coordination through shared code and document repositories, as well as archived chat tools (e.g., Slack). The reluctance to rely on ongoing communication technologies like live chat or video conferencing stems from their geographic and time zone dispersion, as well as the ethos of allowing employees to contribute when and where they feel like doing so (see Srikanth and Puranam 2011, 2014 on generic strategies for coordinating distributed work). The basic template for asynchronous collaboration has a few core elements, such as:

1. A menu of modular tasks: team leaders are responsible for “triaging” (i.e., distributing and allocating) tasks in their teams. Team leaders decompose “issues” (things that need doing or fixing) into tasks to maximize their discreteness and independence from each other (though it is not always possible to accomplish complete separability) and assign a priority level to each of them. Team members then typically self-select tasks.
2. The “git” workflow process: it is a structured process any contribution to the code base and the handbook undergoes. There is no alternative way to edit the code or the handbook than following the “git” workflow process. A clarifying example follows. To get employees familiar with this process which is at the heart of modern software development, one of the first things a new GitLab employee has to do is to add herself to the team page. She has to go into the shared repository containing the team.yml file, which stores information on every GitLab team member. In order to modify the page, the employee needs to (Exhibit 1):
  “Fork” the team.yml file (generate a copy of it),
  Modify it (by adding his name, his job title, his location and to whom he reports),
  Then do a “merge request” (ask a maintainer—a person in GitLab in charge of ensuring the quality of the file- to agree to substitute the old file with the new one).

After an automated technical check to verify that there is no technical incompatibility in the way information was added, the maintainer, by looking at the changes, would decide whether to approve the request. This is the git process needed for every change made to the code base—the repository of code that comprises the GitLab product—and in fact also to the handbook. It represents a process that allows for distributed asynchronous work but also checks for potential coordination failures and clarity on decision rights.

3. The “Minimum Viable Change” principle: since coordination is asynchronous, the risk always exists of coordination failures that would be detected too late (e.g., two individuals working in parallel on the same problem, making one of their efforts redundant, or one making changes that would invalidate the efforts of the other). To minimize this risk, employees are urged to submit the minimum viable change—which could be an early stage, imperfect version of their suggested changes to code or documents. This is expected to serve as a flag to others to make them aware that an employee is working on something to prevent clash or duplication. This is also why iteration is one of the GitLab core values: “We do the smallest thing possible and get it out as quickly as possible”.^6^
4 Communication is kept “asynchronous and public”: employees are encouraged to use means of communications that leave traces and are visible to the broadest set of other employees, and should not necessarily expect a real-time “chat style” interaction. As one of the employees told us, “we do not send internal email here”. Instead, employees can post questions on the Slack channel of the team, and later the team leader can decide whether that information needs to “last”—i.e., be permanently visible to others. If so, it will be stored in a place where the information will be available to everyone in the company, in an “issue” document or on a page in the handbook.
5 Transparent compensation: GitLab publicly shares the compensation calculator it uses to determine employee salary. The calculator computes a salary according to the job role, the level of expertise within the specific job role and a location factor. Each salary is computed using the San Francisco job market as a reference point and adapted to the cost of living and market conditions of the city where the GitLab employee lives. Employees also take stock options—these are assigned to every member of GitLab and pre-determined according to job role, irrespective of location. Each job role has a pre-determined number of stock options (not value). Employees can also be awarded discretionary bonuses for instantiating company values. In the absence of transparency, there may be significant risks of employees in an all remote setting forming inaccurate and perhaps unfavorable beliefs about what others are being paid (e.g., Nai et al. 2020) since there is no natural “corridor gossip”.
6 GitLab members were clear that they are all remote, not flat. They have a fairly traditional looking organization chart,^7^ with managers and team leaders, and also unambiguous task allocation through the concept of Directly Responsible Individual (DRI) (though which task to take on could be self-selected). After (self-)allocation into a taks the DRI has the ultimate responsibility for the completion of a task. The DRI can request and receive (also unsolicited) suggestions by other members of the organization on how to perform her task, but she is free to accept or reject them. Finally, if the output of the task is a contribution to the code or the handbook, it follows the git process. The ultimate approval (i.e., the integration of the output in the codebase) is left to the maintainer of the specific section of the handbook or of the codebase.

#### Generalizability

GitLab’s CEO Sid Sidbrandij argued that in a world of rising national boundaries, visa restrictions and limited talent pools in any one location, GitLab’s model was a source of great advantage: “Access to most of the world talent and no wasted commuting time!” Further, the emergence of the COVID-19 pandemic in early 2020 saw organizations all around the world forced to adopt remote working as part of social distancing measures to mitigate the spread of virus, and GitLab’s approach now seemed like a useful guidepost rather than an oddity. But given the relatively small size of the organization, their ability to recruit enough employees comfortable with working remotely and limited social interaction with colleagues, and the idiosyncratic features of their product (software), could organizations in other sectors learn from their approach?

## All-remote organizations and geographic flexibility

### Prithwiraj Choudhury

The COVID-19 pandemic has forced companies and knowledge workers around the world to adopt remote work. Even prior to the pandemic, however, a new organizational form was emerging, in which the organization had no physical office or physically co-located workers, and all workers—from entry level interns to the CEO—were geographically distributed and working remotely. This organizational form has been described as “all-remote” and a prominent example of this organizational form is GitLab, a company profiled in two recent case studies written at HBS and INSEAD (Choudhury and Salomon 2020; Minervini et al. 2020). Other examples of all-remote organizations include Automattic, Zapier, and Seeq. This commentary will add a few thoughts to Minervini’s introduction to GitLab and will attempt to situate this novel organizational form in light of the prior remote work literature (Cooper and Kurland 2002; Gajendran and Harrison 2007; Bartel et al. 2012; Bloom et al. 2015; Choudhury et al. 2020) and the emerging literature on firm-specific incentives (Kryscynski et al. 2020).

#### An extreme case of geographic flexibility

Choudhury et al. (2020) argue that while traditional work-from-home (WFH) remote work programs, as studied by Bloom et al. (2015) and other scholars, provide the worker with temporal flexibility, granting individuals more control over the hours in which they complete their work (e.g., Briscoe 2007), an alternative form of remote work, which Choudhury et al. (2020) call “work-from-anywhere” (WFA), provides the worker with *geographic flexibility*, i.e., the choice to live in a location (town, city, country) preferred by the worker. To quote Choudhury et al. (2020, pp. 3–4):

We argue that WFA should be viewed as a nonpecuniary benefit that should be preferred by workers who would derive greater utility by moving from their current geographic location to their preferred location. Prior literature in migration and urban studies (e.g., Barcus 2004) has theorized that workers may relocate due to low satisfaction with their current residential location. We theorize that workers self-selecting into WFA and moving from their current location to a more preferred location will experience greater residential satisfaction, greater utility, and based on theorizing by Sauermann and Cohen (2010), will exert greater productivity-enhancing effort. This effect might be especially salient if WFA is perceived by workers as a “firm-specific incentive” (Kryscynski et al. 2020), i.e., an incentive in short supply at other possible employers.

All-remote organizations such as GitLab arguably offer *all* employees geographic flexibility and this is in stark contrast to “hybrid-remote” models, where some workers work remotely while others are physically co-located. Hybrid remote models have been studied in the context of both WFH (Cooper and Kurland 2002; Gajendran and Harrison 2007; Bartel et al. 2012; Bloom et al. 2015) and in the context of WFA (Choudhury et al. 2020).

#### Directions for future research

The emergence of the “all-remote” organizational form raises several interesting questions for organization scholars. I summarize three specific themes:

1. *Professional isolation of remote workers.* Prior research on remote work has extensively studied the topic of professional isolation of remote workers. In the context of hybrid-remote organizations, Cooper and Kurland (2002) document that professional isolation of remote workers is related to (lack of) “employee development activities”, such as interpersonal networking, informal learning, and mentoring. In subsequent literature, Bartel et al. (2012) find that “perceived respect” of remote workers, i.e., the identity-based status perception that reflects the extent to which one is included and valued as a member of the organization, is negatively associated with the degree of physical isolation, and perceived respect mediates the relationship between physical isolation and organizational identification. With the emergence of the all-remote organizational form, given that *all* workers are remote, an interesting question relates to whether the prior patterns of physical isolation and organizational identification of remote workers are now reversed. Another interesting question relates to how all-remote organizations organize interpersonal networking, informal learning, and mentoring. While these activities are important for any organization, as highlighted by Cooper and Kurland (2002) and Gajendran and Harrison (2007), remote workers in hybrid remote organizations have been constrained by the lack of informal mentoring and socialization, including serendipitous face-to-face interactions, and it would be interesting to study whether and how all-remote organizations differ in this regard.
2. *Tacit coordination mechanisms*. Minervini’s introduction of GitLab highlights the asynchronous coordination processes employed by GitLab, and the principle of ‘Minimum Viable Change’ as a means of mitigating coordination failures. Future work could expand on this and study the coordination mechanisms employed by all-remote organizations relative to other distributed organizations. Prior literature (e.g., Srikanth and Puranam 2014) highlight that geographically distributed projects conducted within firms rely extensively on tacit coordination mechanisms (TCMs). It would be interesting to study whether all-remote organizations differ from hybrid remote organizations in developing and/or employing TCMs for minimizing coordination failures.
3. *Management practices around compensation*. Minervini’s introduction outlines compensation policies at GitLab and how wages of the workers are adapted to geography, i.e., the market conditions of the city where the GitLab employee lives. In contrast, Choudhury et al. (2020) document that the United States Patent and Trademark Office in implementing the work-from-anywhere (WFA) policy did *not* adjust wages based on where the employee preferred to relocate. Whether or not to adjust wages based on the location of WFA and all-remote workers has been at the center of a managerial debate for companies such as Facebook (Murphy 2020). While we have an extensive literature on management policies related to setting wages for managers of global firms (Carpenter et al. 2001; Leung et al. 2009), the emergence of the all-remote organizational form raises interesting questions around pay parity and fairness of compensation of workers performing similar tasks distributed around the globe. Future research can shed light on whether policies that adjust wages based on location are more or less optimal compared to policies that do not adjust wages based on the location of the worker.

In conclusion, the emergence of the all-remote organizational form and the provisioning of geographic flexibility to all workers has important implications for research and practice and also raises important questions for organization scholars and scholars of strategic human capital.

## GitLab: tools and work practices for asynchronous collaboration

### Kevin Crowston

GitLab exhibits a fascinating combination of novel organizing features: work from home; radical temporal and geographic distribution, as employees work from around the world on their own schedule; reliance on technology-supported asynchronous collaboration; and widespread transparency. It is important to note that these are separate innovations, each of which could be implemented separately from the others. Indeed, as the case notes, the COVID-19 pandemic has prompted a dramatic overnight increase in work from home, but mostly without changes in the mode of collaboration or degree of transparency.

Of these different innovations, asynchronous collaboration is the one I find most interesting and so is the focus of my commentary (though I did wonder how the company managed employees in different countries, a question that’s addressed in the handbook—answer, it’s complicated). My interest in asynchronous collaboration stems from my research on free/libre open source software (FLOSS) development practices. A lot of the interest in studying FLOSS development was the hope of being able to transfer its advantages to other settings. GitLab makes this transfer quite literally: the company is essentially run like a large FLOSS project. Even the “source code” for the organization, the company handbook, is public (though apparently not all of the actual source code for their products).

What makes this mode of collaboration work? Part of the answer is the tools, which are designed specifically to support asynchronous collaboration. But equally important are the work practices, that is, GitLab (like all organizations) is a socio-technical system requiring interlocking technology and practices. The practices that enable asynchronous work are well described in the case, such as decomposing work into pieces that don’t have many dependencies, thus reducing the need for more intensive coordination that would benefit from or require real-time interaction. GitLab also adopts the FLOSS practice of (mostly) allowing team members to self-select tasks, further reducing the need for coordination to decide who does what.

Asynchronous work further benefits from the possibility of stigmergic coordination, meaning coordination through a shared work product rather than through direct communication. In other words, if I can see what you are doing, I may not have to talk with you about it, at least not as much. Crowston et al. (2019) identified three socio-technical affordances that support stigmergic coordination, all of which can be seen in the GitLab case.

First, the contributions to the shared work must be visible to other contributors. In GitLab (as in FLOSS development) visibility is supported by the GitHub technology. But equally important are the social practices that ensure that work is visible, such as the “minimum viable product” concept that makes outputs available early in their development (in FLOSS this principle is stated as “commit often and early”). The emphasis on communication that “leaves traces” that others can review again promotes visibility of work. A question I had in this regard was how workers find what’s relevant to them in the mass of documentation. As the case notes, lasting information can be pinned or added to the handbook, but finding a relevant other project in a mass of work contributions could be a challenge.

The second affordance is legibility, meaning that workers can interpret others’ contributions to the shared work product as cues for their own work. In part, legibility is structured by the system, which has specific types of contributions (e.g., pull requests) that demand specific kinds of responses. But as well, this affordance requires development of shared understandings about the meaning of different contributions—no small trick in a distributed environment. These understanding are developed and reinforced both by formal documentation (the handbook) and by seeing contributions and the discussions around them in the form of electronic records. A question here is what kind of distributed mentoring can be provided to new employees to help them quickly develop accurate mental models of the work. Given the similarity of this mode of collaboration to FLOSS development, I suspect one approach is to hire people with that experience.

The final affordance is combinability, meaning that contributions have to be combinable with those of others to form a coherent product. Again, the technical infrastructure helps integrate different contributions, e.g., a pull request to update some part of a document. But equally important are the social practices around making contributions in the forms of updates to documents (the “git process”).

Exploring the factors that enable GitLab’s approach to asynchronous collaboration helps set boundary conditions on this form, i.e., to identify where it might not work. A document-centric approach works for GitLab because it is a software company that runs on documents of different sorts. Asynchronous work might not work for physical goods that require co-presence to manipulate. For instance, it is (currently) hard to work in a factory from home or to deliver a baby. Moving pianos cannot be done one-third at a time. Second, the visibility of work needed for stigmergic coordination might be problematic in settings where some information or tasks cannot be shared, e.g., patient notes in a doctor’s office or plans for new product development (though it could still work within a smaller group; even at GitLab, not all information is shared). Finally, as the GitLab handbook notes, a workplace with limited social engagement is not for everyone.

Acknowledging these boundaries, the innovations of GitLab still have broad implications. Knowledge work is largely document-based and the on-going digitization of the economy makes this mode ever more relevant. COVID will likely be with us for some time, so many workers will not be back in the office soon (or indeed, ever). The case describes a radical implementation of technology and work practices across an entire organization. But these innovations can also be applied individually and to smaller units to achieve the benefits of reduced coordination cost on a smaller scale. In that regard, the GitLab case provides a useful model for all managers.

## Challenges of an all-remote company

### Linus Dahlander

I grew up in the Swedish countryside in a tiny village that I usually describe as having more cows than people. My childhood was a bit of an Astrid Lindgren book. My parents are part of the generation where many attended university and then moved to rural parts of the country to have a life close to nature. It worked for my parents as my dad is a doctor and my mom a teacher who can always find work independently of where you move. I moved to the city when I started attending university and have lived in cities ever since. It would be difficult for me now to live in the village where I grew up, even if I like the idea. I have to be present at the workplace and the personal interactions are important for me to know the inner workings of the organization. What I would like is to work remotely, so I can live in the countryside again. But even if I would like remote work to be an effective reality, so I could spend my time in the remote Swedish countryside, I think there are several scope conditions where this model works better than in others. Minervini’s fascinating tale of GitLab reminds me of some challenges pointed out in earlier literature and calls for more empirical work to address some challenges that can arise.

#### Different types of autonomy

The GitLab example reminds us how important it is to consider autonomy and its effects on employee engagement and innovation (Amabile and Gitomer 1984). Many other examples we study in novel forms of organizing (Puranam et al. 2014) provide autonomy in a broad sense of allowing the choice of tasks and often collaboration partners—at least for a certain proportion of time. The GitLab example reminds me of Bailyn’s (1985) early qualitative work that distinguishes between strategic autonomy (what to do) and operational autonomy (how to do it). Her point is important in that she proposes that engineers and scientists would like operational autonomy about how to pursue a task, but not have freedom on what to work on. Conversely, she speculates that managers seek more strategic autonomy. Perhaps this is a bit too simplistic, but her early observations remind us about the importance to consider for what kinds of people autonomy is desirable and whether they can use it effectively. Autonomy is a double-edged sword. On the one hand, it may increase loyalty, engagement, and creativity. On the other, it can create coordination problems and people may let complacency take precedence over choosing the best collaborators and tasks to work on. The GitLab example is fascinating in that they seem to grant more operational autonomy to employees and avoid some challenges of allowing strategic autonomy.

In a recent paper, we used a randomized control trial to study how granting students autonomy in choosing ideas vs collaborators in a course (inspired by the lean start-up ideas) affects pitch-deck performance. We found that granting autonomy in choosing ideas outperform the other conditions, which implies that people picked worse collaborators than random (see also Clement and Puranam 2018 for a modeling paper along similar lines). Connecting this to GitLab, it is important to consider different types of autonomy. The GitLab case seems to be a case in the middle: There is no full autonomy but rather operational autonomy. While we know about different types of autonomy, we are more in the dark when it comes to heterogeneous treatment effects from more experimental setups about the desire for autonomy and whether it is used appropriately.

#### Observable inputs and team work

The transparent compensation reminds me of the field experiment by Card et al. (2012). They studied the effect of disclosing information on peers’ salaries on job satisfaction and job search intentions. This study randomly informed some employees of the University of California about a new website listing University employees’ salaries. Workers below the median of their unit reported lower satisfaction, whereas those above were unaffected. It seems like GitLab somehow overcomes the “salary taboo” present in many organizations, but there could be challenges in other settings.

Transparent compensation works well in situations where individual contributions are traceable, possible to observe and immediately connected with the individual. Striking a similar chord as GitLab, my son talks about tryouts for Fortnite clans where “interviews” are conducted and where the kids’ building and aiming skills are put to a test. In some ways these kids beat HR directors by getting deep insights into someone’s skill-level. How would this work when an employee's contribution is harder to observe? An added challenge is that of decomposing individual effort to a team. Tasks are not always decomposable. In the study mentioned above on teams (Boss et al. 2020), we asked all the team members after the 11-week project what percentage they contributed. When summing up to the team, all teams had over 100%… This finding is not new—people tend to think higher of their own work than others do. But in other distributed settings this is an obstacle for forging team collaborations where money is involved. For instance, this was one reason many of the prize competition companies such as InnoCentive struggled with introducing teams.

#### The formation of a company culture

GitLab takes pride in being a company with no email communication, and where they use Slack as asynchronous communication, and save some correspondence for the future. The obvious challenge in an all-remote company is to build a culture and sense of belonging. That said, GitLab also organizes other ways to connect and build a culture. They organize “daily team calls, regional co-working days, virtual “watercooler” chats to get to know each other on a more personal level, general group conversations, "ask me anything" conversations with our leaders, and GitLab Contribute.” GitLab Contribute is a yearly summit where the whole company comes together in a different city. While being a remote company on a daily basis, they have many mechanisms in place to instill a company culture. This also resembles what some open source communities have done with asynchronous communication through mailing lists and code repositories and events where core contributors come together to meet. In a distributed all-remote company, these events strike me as important to create a shared understanding. As the Covid-19 pandemic struck, we as academics have experienced something similar with people working remotely. The video-conferencing and our equivalents of “watercooler” chats are important to keep up to speed. An open question in all remote companies is how to create distant ties between previously disconnected people. Virtual means of communication are important to maintain and deepen existing ties but often struggle to create distant ties that are more sustainable. This is where I personally believe the Summit makes sense. The lesson for academics is that even an all-remote company sometimes needs colocation.

#### Selection at the gate and by performance

I have previously studied open source communities that resemble many of the organization features described by Minervini. For instance, my past work on GNOME shows how a widely geographically distributed community of peers self-organize. They use similar principles of controlling interdependencies and check-ins of work. In that case, some peers had more power than others in controlling work, but had no direct authority to tell others what to do. There are obvious differences to Minervini’s case in that there are employment contracts in place. Another difference is that an open source community can afford double or triple work to a much greater extent—inefficiencies are acceptable when you don’t pay for them.

It begs the question about the importance of selection at the gate—who gets hired by GitLab to begin with? Would it work in other contexts where there is a wider distribution of talent or even work ethic? “Self-organizing” companies such as Valve or Zappos go to great lengths to make sure they get the right people with a cultural fit able to work independently under great autonomy. The open compensation structure would potentially sort out underperforming people at GitLab as they cannot hide in the shadow of a high-performer. But if people get selected strongly to begin with, then it suggests that it works for a smaller set of individuals than we perhaps think. This remains an open and exciting research question.

## GitLab: a human resource management perspective

### Sumita Raghuram

Many companies, especially startups, start as “all remote” organizations (e.g., Mozilla, Verifone, Scopic, Articulate) even though at some later stage they move to a ‘bricks and mortar’ structure. The all remote structure enables nimble operations by staffing employees from all over the world and taking advantage of time zone differences. GitLab too is an all remote organization and like several others, it operates in the software development industry. There are some unique organizational practices that work in favor of GitLab’s virtual structure.

#### What works for GitLab

First, it has a very high level of transparency inside and outside the company. For example, employees are encouraged to use means of communications that leave traces and are visible to the broadest set of other employees. In addition, GitLab has the norm of not sending internal emails. Instead, employees can post questions on the Slack channel of the team where the information is available to everyone in the company. Second is the emphasis on equitable and fair compensation–something that is universally desirable by employees. In this regard, GitLab’s use of a compensation calculator to determine employee salary is particularly beneficial. The calculator computes a salary according to the job role, the level of expertise within the specific job role and a location factor, such that it incorporates the cost of living and market conditions of the city of employees’ residence. Third, the company handbook documents its formal organizational structure and processes. The handbook is public and anybody can view it inside or outside the company. Such transparency is important to note because most of the research points to the fragility of trust in virtual teams and virtual organizations (Cascio 2000). One of the predictors of trust is a high level of openness and transparency in organizational practices—and GitLab has worked hard to ensure it is in place. Lack of face-to-face interactions coupled with lean communications media reduces opportunities to seek clarifications or feedback. This can lead to misattributions and misunderstandings among people. It can then escalate to counterproductive conflictual situations.

A second set of practices at GitLab are oriented towards motivating its employees. These include GitLab’s use of stock options and discretionary bonuses that tie employees’ pay to performance. This is directly related to the principles of the expectancy theory. In addition, the employees’ jobs are characterized by a high level of autonomy. GitLab has consciously worked towards creating asynchronous coordination and collaboration through shared code and document repositories, as well as archived chat tools. This allows employees to contribute when and where they feel like working which frees them up from the need to be tied to another. This is of course specific to the nature of work in that it allows for the possibility of task independence, or relatively easy ways to manage interdependence. Coordinating interdependent tasks can lead to less autonomy. As such GitLab’s motivational practices are consistent with what knowledge workers would typically expect, i.e., to be recognized for their performance and to be able to perform when and how they want to with minimal supervision (Horwitz et al. 2003).

#### Possible challenges for GitLab

Running a virtual organization where all employees are remote can be challenging in many ways. These challenges include building a cohesive organization culture, selection and onboarding of new employees, and maintaining a healthy work-life balance. As GitLab continues with its current mode of operation, these are some of the issues that might need attention.

Research demonstrates that cohesive organizational culture in form of organizational identification can suffer when employees are not in constant contact with the visible signs and symbols of their organization (e.g., the building and other artifacts connected with their organization; the presence of their supervisors and peers) (Wiesenfeld et al. 1999). The remoteness can present itself as a centrifugal force, distancing employees from the organization’s core. In absence of constant reminders of the core values of the (virtual) organization, the leaders can lose the ability to consistently communicate these. Organizations therefore have to be mindful of the frequency, content and mode of communication with the employees. Lack of synchronous communication can further exacerbate the weak connectivity across time zones. While GitLab holds a yearly “Contribute” event, an informal corporate get-together for GitLab team members, it may or may not be adequate enough to build community and reinforce cultural values especially as the organization grows in size and locational distributions.

The second challenge is with regards to hiring, onboarding and firing at GitLab which takes place remotely. With respect to hiring GitLab may want to consider individual differences in people’s ability to work from remote (e.g., their ability to structure work days, their need for affiliation, or their ambiguity tolerance levels) (Wiesenfeld et al. 2001). These are important characteristics that can make an employee successful in a remote work context. Hiring and onboarding require organizational contextualization for newcomers. It can be rather disconcerting for new individuals to understand how they can get socially integrated with their co-workers or their supervisors. Newcomer socialization is the process whereby newcomers also learn the behaviors and attitudes necessary for assuming roles in an organization (Van Maanen and Schein 1979). Individuals experience ambiguity about both job requirements and their social integration. Traditional scholarly treatment of socialization presumes face to face interactions among organizational members or use of paper documents. Newcomers’ reliance on electronic communication channels alone for socialization may pose challenges in reducing the ambiguity. Mentoring can be very rewarding in socialization of newcomers but difficult to carry out if not done through informal meetings which are possible only in a face to face setting. Even firing may require a human/humane touch so as to demonstrate fairness or justice. It is in the interest of the company to maintain its image and reputation as a ‘good place to work’.

Finally, remote work typically involves working from home. This is especially likely to be the case for GitLab which lacks any physical work location. While work from home carries benefits such as greater autonomy with respect to time and place of work, it also blurs the boundaries between work and personal lives (Raghuram and Wiesenfeld 2004). The constant use of mobile technologies to stay connected to work during personal time is a strong temptation and can result in emotional exhaustion (e.g., Lanaj et al. 2014). The presence of nonwork cues might make it more difficult for employees to focus on work. These effects are particularly noticeable for employees with young children or those with inadequate work space in their homes. Lack of clear and consistent boundaries between work and personal lives can lead to increased conflict between work and nonwork and stress, and thereby overall reduced job satisfaction.

## Conclusions

### Marco Minervini

Can asynchronous all-remote collaboration become a widely diffused practice? What are the boundary conditions for scaling and for its application outside of GitLab and outside of software development?

My goal was to stimulate a debate on whether a rare species, such as GitLab, could diffuse to the point of becoming widespread, especially after the emergence of a facilitating factor such as COVID-19.

I want to thank the commenters for their time and insightful comments. Their different disciplinary perspectives significantly helped in extending the understanding of the mechanisms that allow GitLab and the all remote asynchronous mode of working to work at the scale of more than 1000 employees fully distributed across all time zones and the challenges that the model can face in scaling up and diffusing outside software development. In the following paragraphs I will highlight some of these challenges.

#### All remoteness, organizational identification and shared norms

The commenters argued that the absence of colocation could hinder organizational identification and the development of common mental models. Raghuram warns that the absence of contact with visible signs and symbols of the organization (e.g., artifacts and leaders) could hinder organizational identification, which is instrumental for coordination and motivation. In addition, Choudhury cites the consistent evidence on the negative effect of working remotely on organizational identification, but raises an interesting and important question, the answer to which is fundamental to understand the wide sustainability of the all remote model: are the undesirable effects of working remotely on organizational identification still present in *all* remote companies?

In understanding the implications of all remoteness on organizational sustainability and performance through organizational identification, I believe we need to distinguish between the implications of all remoteness on individual motivation and on collective coordination.

For individual motivation, consistent evidence suggests that organizational identification leads to more work effort, willingness to perform extra role behaviors, and task performance generating benefits for the organization (Riketta 2005). While existing studies show a lower level of organizational identification by remote workers, the degree to which the level of organizational identification is driven by intensity of exposure to organizational symbols or by differences in exposure compared to the other members at who work collocated is an open question. If organizational identification is weakened by inequality of exposure [through, as suggested by Choudhury, a mechanism like perceived respect (Bartel et al. 2012)] more than mere intensity of exposure, we might expect a lower impact of remoteness on average organizational identification in all remote organizations.

For coordination through shared mental models and norms, research suggests that the most conducive organizational cultures are “the ones with both a high level of consensus about the systems of norms and intensity around the most valued norms” (Chatman et al. 2014, p. 786; Marchetti 2019). All remote companies à la GitLab seems to be uniquely positioned in clearly defining a common set of valued norms and “broadcasting” them without mediation to its sparse employees. GitLab’s extensive onboarding process as well as the presence of a handbook that acts as a “single source of truth” are two examples of single source of definition of organizational norms. However, a focused culture becomes effective only to the degree it is embraced by employees. As highlighted by Raghuram, the degree to which all remote companies will be able to reinforce cultural values in the absence of exposure to physical symbols and through limited reliance on social pressure by superiors and peers remain an open question. GitLab is careful to address the two abovementioned challenges. It attempts to substitute physical symbols with virtual ones, for instance by devising ad hoc emoji for each of their codified cultural values and substitutes the reliance on informal social pressure to enforce company culture with the explicit inclusion of respect of cultural values as criterion in decisions on promotions, compensation and firing. Questions may arise on the degree to which such highly codified and explicit culture could be sufficiently comprehensive (e.g., inclusive of non-task related norms) and adaptive to shocks.

#### Negative feedback loops in the diffusion of all remote companies

The authors’ comments also suggest that the diffusion of this organizational species could be hindered by a negative feedback loop in the number of all remote companies, driven by two alternative mechanisms: (1) a reduction in the ability to adequately select a fitting workforce given a fixed stock of remote-fit workers; (2) the dilution of the motivational benefits derived from employees’ perception of the possibility to work remotely as a firm specific incentive, once this possibility becomes highly diffused.

The former mechanism works as follows. As highlighted by Dahlander, the direct and indirect challenges posed by absence of colocation can be addressed, as in many other unusual forms of organizing, through a thorough and careful selection of personnel. However, Raghuram and Crowston both stress that working remotely is not made for all. Not everyone has a domestic environment conducive for working remotely or have the correct skills, attitudes or preferences for effective remote work. Hence, a core boundary condition for the diffusion of all remote companies is the very existence of a labor supply that would grant the possibility to companies to perform the accurate selection needed to support such ways of working. Given a fixed stock of the labor force that is fit for remote work, there is a threshold level of the number of company that could be all remote. Any excess in the number of all remote companies would hinder the ability of all of them to perform the needed selection to support their unusual form of organizing, leading to the death or transition to another model of (at least) the companies in excess, generating a negative feedback loop around the threshold.

At the moment, the portion of the workforce that is actually fit for working fully remotely in the long run is mostly unknown. In absence of this information the degree of feasible diffusion of the all remote model remain unknown.

Choudhury suggests an alternative mechanism that would similarly generate a negative feedback loop in the diffusion of all remote companies, placing a cap on it. Given its current rarity, the possibility to work remotely could be seen as a firm specific incentive, spurring additional efforts from employees (Kryscynski et al. 2020). These additional effort can (at least) compensate for the challenges of working all remotely. However, the more diffuse the availability of all remote jobs, the less firm specific the incentive will be perceived, reducing its ability to spur higher efforts.

#### Transparency acceptance as boundary condition

Crowston highlights an additional boundary condition for the expansion of the all remote and asynchronous GitLab model. As it extensively relies on transparency, the model works in contexts where risks generated by transparency are particularly low. All remote and asynchronous companies have particularly high costs of information omission. Absence of information needed to perform a task takes more time to be compensated in remote and (especially) asynchronous organizations. Hence, full transparency of information is a strategy to minimize risks of information omission errors and their consequent costs. However, this strategy is viable only when risks and costs of information commission errors are particularly low or absent, i.e., where making information available to anyone implies low risk or cost (be it competitive or legal) for the organization. What GitLab (especially before becoming a public company) and FLOSS have in common, for different reasons, is very low levels of perceived risks and costs of commission errors. Other organizations, like the health care organizations mentioned by Crowston, are at the exact extreme opposite in terms of costs and risks of commission errors, due to privacy concerns. In their case, full transparency by default is not a viable option and implementing a complex mode of selective transparency could be extremely costly. Hence, the scope condition for the diffusion of an all remote asynchronous model is driven by the costs of information commission errors that companies would face in implementing full transparency of information. In other words, how many organizations out there can afford to minimize information omission errors by granting unconditional transparent access (at least) to their internal members?

To sum up, while it is clear that the GitLab asynchronous all remote model will not be applicable for every organization, our understanding of the degree to which the model can diffuse is strongly dependent on currently unanswered empirical questions. Can the homogeneity granted by an all remote model reduce the historical limitations of hybrid remote models? What is the proportion of workers that is fit for remote work? Which organizations can sustain a high level of transparency and diffusion of information? Can the model be applied on portions of an organization (specific teams or division), as suggested by Crowston, or is the model sustainable only if applied unconditionally across it? These are all questions we need to answer as organization design scholars to support current managerial considerations on transitioning to remote models spurred by the COVID-19 shock.

## Data Availability

The datasets generated and/or analyzed during the current study are not publicly available as the data format (video) cannot ensure participants’ anonymity and confidentiality.

## Footnotes

1 https://web.archive.org/web/20181030162842/https://about.gitlab.com/company/team/

2 https://about.gitlab.com/stages-devops-lifecycle/

3 https://www.forrester.com/report/The+Forrester+Wave+CloudNative+Continuous+Integration+Tools+Q3+2019/-/E-RES148217#

4 https://about.gitlab.com/handbook/

5 https://about.gitlab.com/company/culture/contribute/; Participation to the contribute event is not mandatory for GitLab employees, but it is highly encouraged. Latest contribute events had an attendance of about 85% of GitLab employees.

6 https://about.gitlab.com/handbook/values/#iteration; https://about.gitlab.com/handbook/values/#make-small-merge-requests

7 https://about.gitlab.com/company/team/org-chart/
